# Supplementary material for: Environmental conditions alter behavioural organization and rhythmicity of a large Arctic ruminant across the annual cycle
Source: R Soc Open Sci. 2020 Oct 28;7(10):201614. doi: 10.1098/rsos.201614 (PMC7657931; doi:10.1098/rsos.201614)
Supplement: Supporting Figures including study area, results of HMM modelling and monthly periodogram results [file rsos201614supp1.docx]

**Supporting Information for:**

Environmental conditions alter behavioural organization and rhythmicity of a large Arctic ruminant across the annual cycle

Floris M. van Beest^1,2,*^, Larissa Teresa Beumer^1^, Marianna Chimienti^1^, Jean-Pierre Desforges^1,3^, Nicholas Per Huffeldt^1,4^, Stine H. Pedersen^5,6^, Niels Martin Schmidt^1,2^

^1^ Department of Bioscience, Aarhus University, Frederiksborgvej 399, 4000 Roskilde, Denmark

^2^ Arctic Research Centre, Aarhus University, Ny Munkegade 116, 8000 Aarhus C, Denmark

^3^ Natural Resource Sciences, McGill University, Ste Anne de Bellevue, Quebec H9X 3V9, Canada

^4^ Greenland Institute of Natural Resources, 3900 Nuuk, Greenland

^5^ Department of Biological Sciences, University of Alaska Anchorage, Anchorage, United States

^6^ Cooperative Institute for Research in the Atmosphere, Colorado State University, Fort Collins, Colorado, United States

* Corresponding author: flbe@bios.au.dk

**
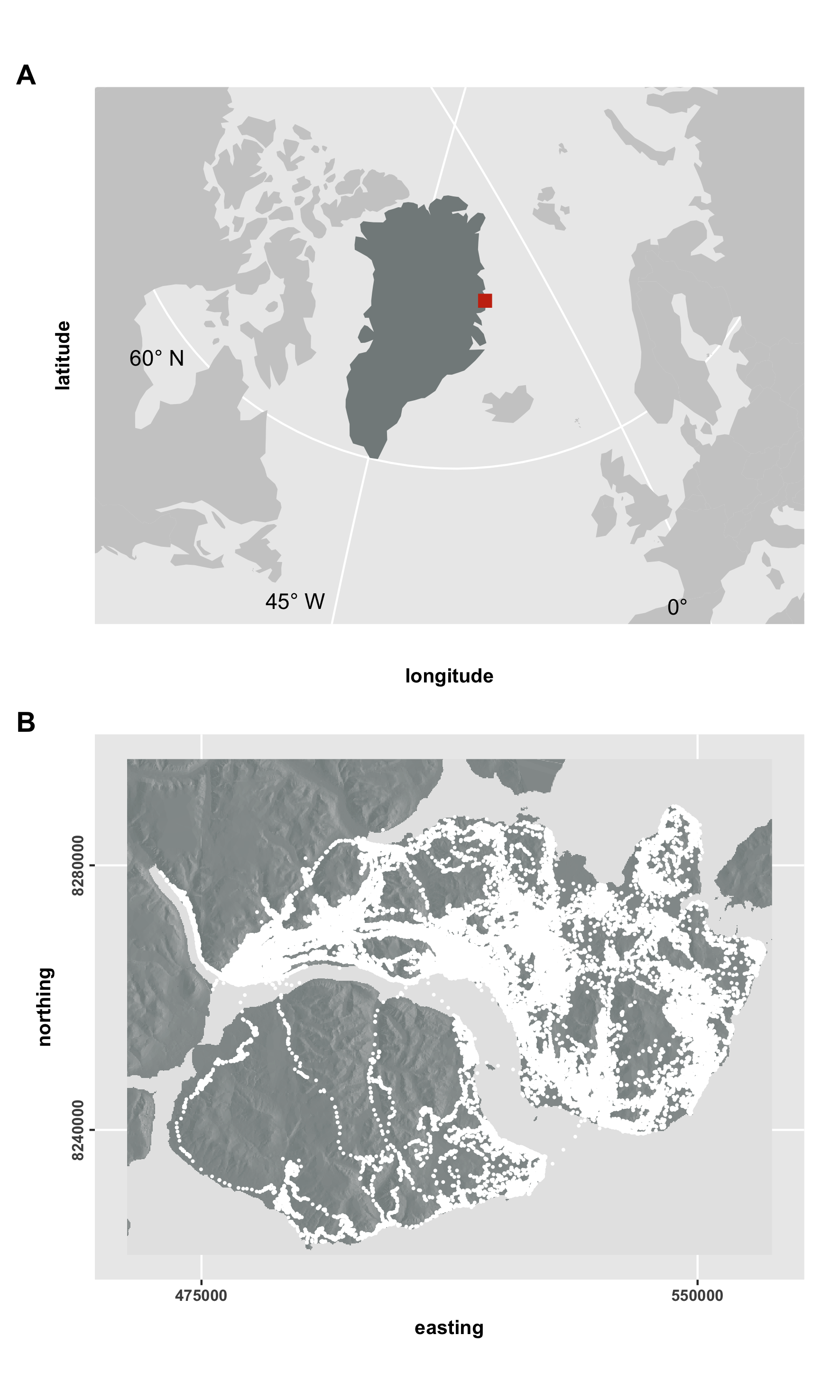

Figure S1**. Panel A shows the location of the study area (Zackenberg) in north-east Greenland (red square). Panel B shows all GPS locations (N=242 378) of 19 adult female muskox individuals used in the analyses.

**
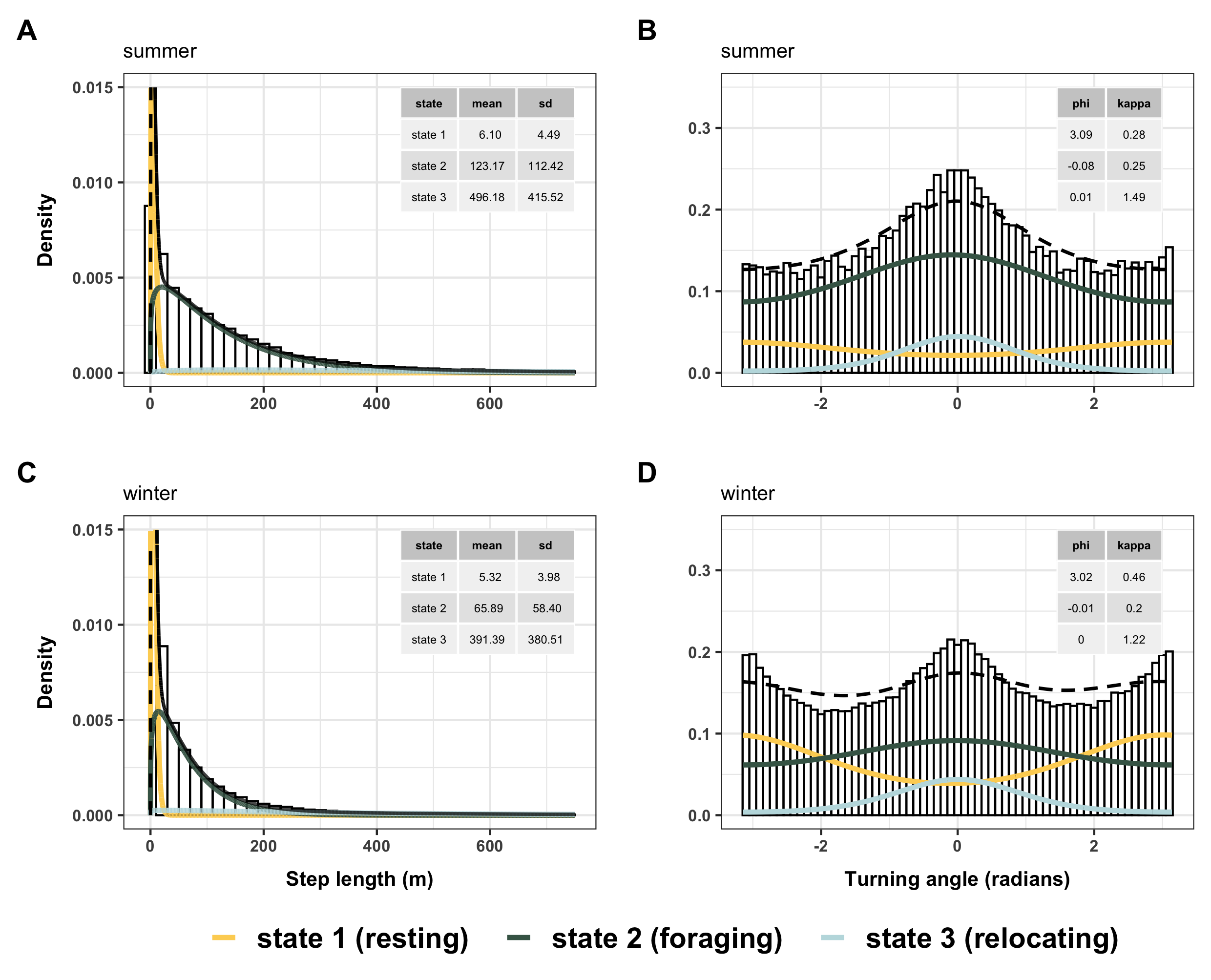
**

**Figure S2.** Histograms of step length and turning angle, respectively, for the summer (A, B) and winter (C, D) season, overlaid with the state-dependent distributions as estimated by the HMMs. The state-dependent distributions were weighted according to the proportion of time spent in the different states, as inferred by the Viterbi sequence. Dashed black lines indicate the associated marginal observation distributions. Note that the x- and y-axes for step length were truncated at the upper range limit to facilitate visualization (maximum observed step length was 3486 m for summer, and 3897 m for winter). Tables included in panels provide parameter estimates per state and model (mean step length with standard deviation; mean turning angle (phi) and angle concentration (kappa)).

**
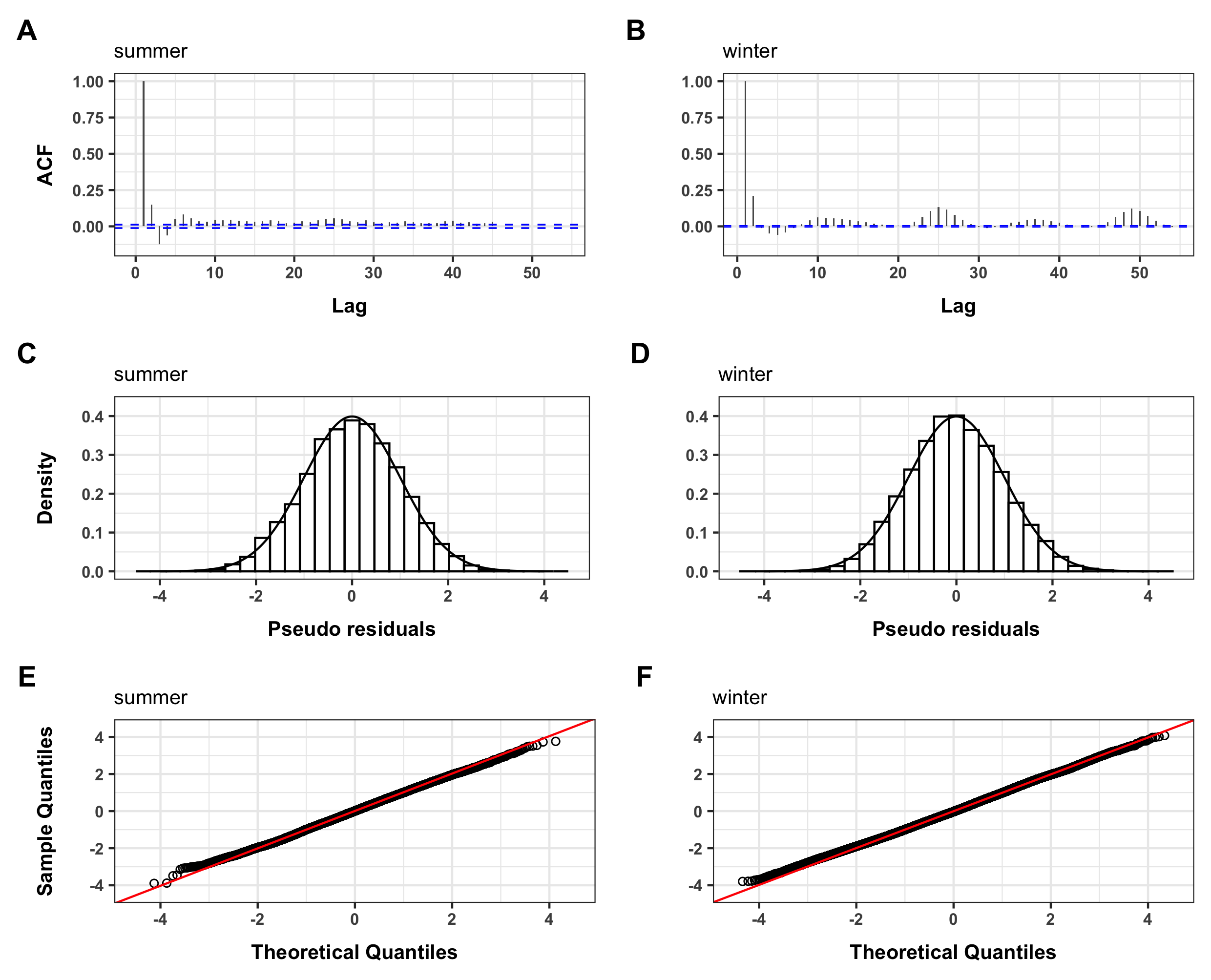
**

**Figure S3.** Autocorrelation structure (A, B), distribution (C, D) and quantile-quantile (E,F) plots of pseudo-residuals for the movement variable step length included in the three-state HMMs fitted to the muskox movement data for the snow-free summer (A, C, E) and the snow-covered winter (B, D, F) season.

**
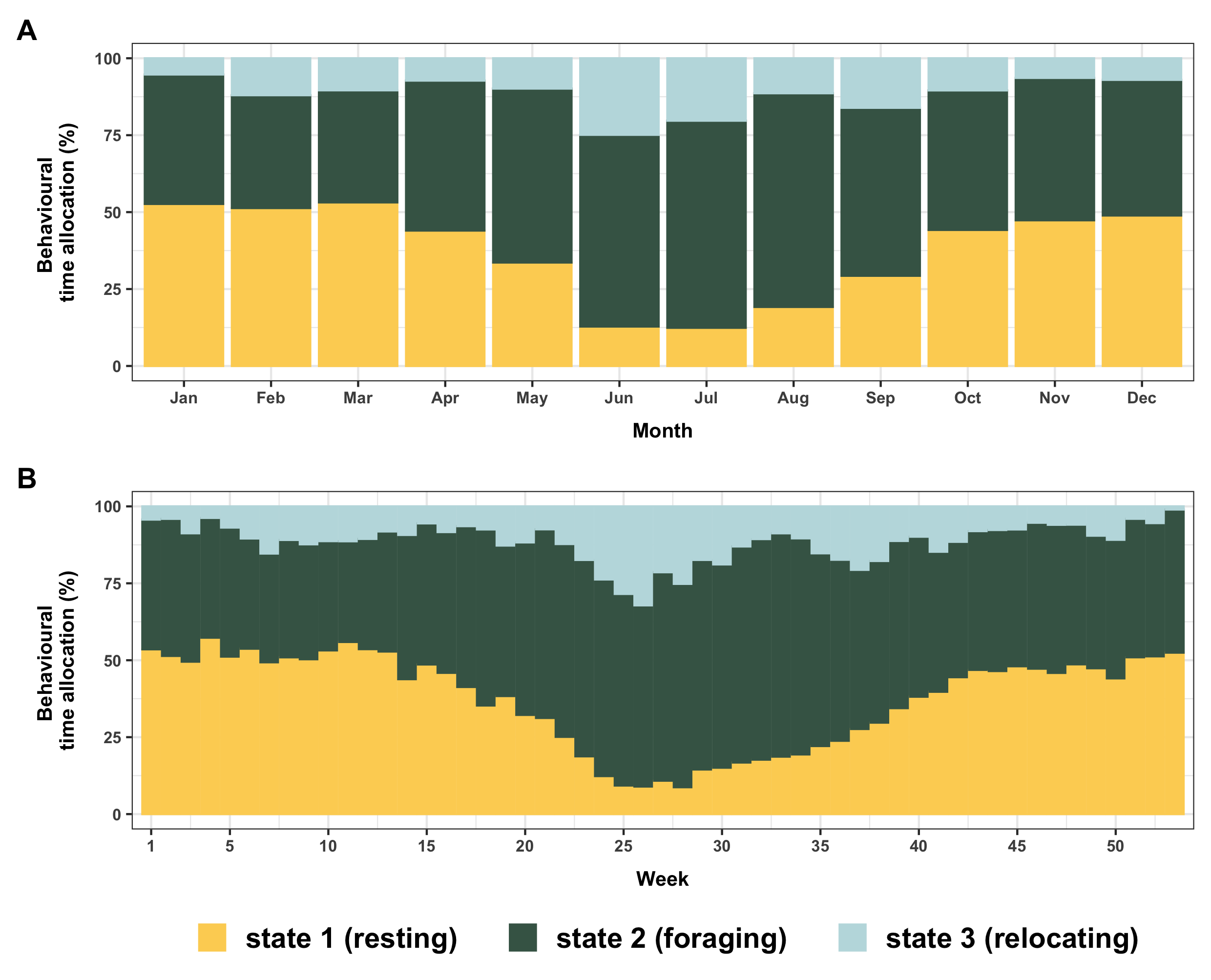
**

**Figure S4.** Behavioural state activity budget across months (A) and weeks (B) for all individuals pooled.

**Figure S5.** Proportion of adult female muskox in each behaviour-based rhythm class as determined by Lomb-Scargle periodogram analysis on a monthly time-scale. Rhythm classes included: ultradian (peak detected in period 2 h–18 h only), circadian (peak detected in period 18 h–36 h only), ultradian & circadian (peaks detected in period 2 h–18 h and 18 h–36 h), or arrhythmic (no significant peak detected). Photoperiod (hours of daylight) over the entire year is plotted on top of each panel as light grey.

**Figure S6.** Plot of the monthly-scale predicted probability of being rhythmic in the foraging-, resting-, and transit-state and for net displacement as a function of photoperiod (h of daylight), snow depth (m) and NDVI (index 0-1). Solid lines show the mean predicted value with shaded grey areas representing the 95% confidence interval, which were generated using a bootstrap procedure with 100 simulations. Non-significant (p>0.05) relationships are plotted without 95% confidence intervals. Predictions for each relationship were made while keeping other variables in the model (Table S1) constant at their mean value.

**Table S1**. Output of the monthly-scale generalised mixed effect models predicting the probability of rhythmicity in adult muskox females as a function of environmental covariates. Separate models were run for each behavioural state and for the net displacement metric. Mean estimate and the 95% confidence interval (calculated with a bootstrap simulation procedure) are provided for each covariate. Confidence intervals that did not overlap with 0 were considered evidence for statistically significant relationships.

|  | **Foraging** |  |  |
| --- | --- | --- | --- |
| **Variable** | Estimate | CI low | CI high |
| (Intercept) | 1.42 | 1.055 | 1.792 |
| Elevation (m) | 0.202 | -0.176 | 0.580 |
| Dense veg. habitat (prop) | -0.103 | -0.379 | 0.172 |
| Snow depth (m) | 0.087 | -0.278 | 0.453 |
| NDVI (index 0-1) | -0.485 | -0.831 | -0.140 |
| Photoperiod (h) | -1.155 | -1.564 | -0.745 |
|  | Var | Std. Dev. |  |
| ID/year (random intercept) | 0.492 | 0.703 |  |
|  |  |  |  |
|  |  |  |  |
|  | **Resting** |  |  |
| **Variable** | Estimate | CI low | CI high |
| (Intercept) | 5.096 | 3.310 | 6.882 |
| Elevation (m) | -0.257 | -0.907 | 0.392 |
| Dense veg. habitat (prop) | -0.285 | -0.787 | 0.217 |
| Snow depth (m) | 1.664 | 0.927 | 2.401 |
| NDVI (index 0-1) | 0.480 | -0.069 | 1.029 |
| Photoperiod (h) | -6.561 | -8.543 | -4.579 |
|  | Var | Std. Dev. |  |
| ID/year (random intercept) | 0.532 | 0.728 |  |
|  |  |  |  |
|  | **Transit** |  |  |
| **Variable** | Estimate | CI low | CI high |
| (Intercept) | 1.570 | 1.272 | 1.869 |
| Elevation (m) | 0.192 | -0.142 | 0.526 |
| Dense veg. habitat (prop) | -0.178 | -0.456 | 0.101 |
| Snow depth (m) | -0.377 | -0.735 | -0.020 |
| NDVI (index 0-1) | 0.062 | -0.353 | 0.478 |
| Photoperiod (h) | -0.026 | -0.333 | 0.281 |
|  | Var | Std. Dev. |  |
| ID/year (random intercept) | 0.001 | 0.001 |  |
|  |  |  |  |
|  | **Displacement** |  |  |
| **Variable** | Estimate | CI low | CI high |
| (Intercept) | 2.838 | 1.936 | 3.741 |
| Elevation (m) | 0.003 | -0.543 | 0.548 |
| Dense veg. habitat (prop) | -0.328 | -0.749 | 0.093 |
| Snow depth (m) | 1.179 | 0.595 | 1.763 |
| NDVI (index 0-1) | 0.406 | -0.065 | 0.876 |
| Photoperiod (h) | -4.810 | -6.015 | -3.605 |
|  | Var | Std. Dev. |  |
| ID/year (random intercept) | 0.001 | 0.001 |  |

**Table S2**. Output of the weekly-scale generalised mixed effect models predicting the probability of rhythmicity in adult muskox females as a function of environmental covariates. Separate models were run for each behavioural state and for the net displacement metric. Mean estimate and the 95% confidence interval (calculated with a bootstrap simulation procedure) are provided for each covariate. Confidence intervals that did not overlap with 0 were considered evidence for statistically significant relationships. The models form the analytical basis for Figure 3 in the main article.

|  | **Foraging** |  |  |
| --- | --- | --- | --- |
| **Variable** | Estimate | CI low | CI high |
| (Intercept) | 2.257 | 2.032 | 2.481 |
| Elevation (m) | 0.257 | -0.026 | 0.488 |
| Dense veg. habitat (prop) | -0.105 | -0.258 | 0.049 |
| Snow depth (m) | 0.010 | -0.200 | 0.221 |
| NDVI (index 0-1) | -0.399 | -0.568 | -0.230 |
| Photoperiod (h) | -0.790 | -1.001 | -0.578 |
|  | Var | Std. Dev. |  |
| ID/year (random intercept) | 0.011 | 0.105 |  |
|  |  |  |  |
|  |  |  |  |
|  | **Resting** |  |  |
| **Variable** | Estimate | CI low | CI high |
| (Intercept) | 3.507 | 3.001 | 4.012 |
| Elevation (m) | -0.026 | -0.262 | 0.210 |
| Dense veg. habitat (prop) | -0.117 | -0.288 | 0.054 |
| Snow depth (m) | 1.028 | 0.776 | 1.279 |
| NDVI (index 0-1) | -0.062 | -0.255 | 0.131 |
| Photoperiod (h) | -3.069 | -3.530 | -2.607 |
|  | Var | Std. Dev. |  |
| ID/year (random intercept) | 0.001 | 0.001 |  |
|  |  |  |  |
|  | **Transit** |  |  |
| **Variable** | Estimate | CI low | CI high |
| (Intercept) | 1.779 | 1.611 | 1.948 |
| Elevation (m) | 0.144 | -0.034 | 0.323 |
| Dense veg. habitat (prop) | -0.175 | -0.363 | 0.013 |
| Snow depth (m) | -0.197 | -0.328 | -0.066 |
| NDVI (index 0-1) | 0.003 | -0.208 | 0.214 |
| Photoperiod (h) | 0.262 | 0.102 | 0.423 |
|  | Var | Std. Dev. |  |
| ID/year (random intercept) | 0.008 | 0.092 |  |
|  |  |  |  |
|  | **Displacement** |  |  |
| **Variable** | Estimate | CI low | CI high |
| (Intercept) | 2.176 | 1.906 | 2.445 |
| Elevation (m) | -0.054 | -0.254 | 0.146 |
| Dense veg. habitat (prop) | -0.172 | -0.321 | 0.023 |
| Snow depth (m) | 0.662 | 0.459 | 0.865 |
| NDVI (index 0-1) | 0.003 | -0.167 | 0.173 |
| Photoperiod (h) | -2.020 | -2.286 | -1.755 |
|  | Var | Std. Dev. |  |
| ID/year (random intercept) | 0.001 | 0.001 |  |
